# Evaluation of an Innovative Method for Calculating Energy Intake of Hospitalized Patients

**DOI:** 10.3390/nu8090557

**Published:** 2016-09-09

**Authors:** Sheila Cox Sullivan, Melinda M. Bopp, Paula K. Roberson, Shelly Lensing, Dennis H. Sullivan

**Affiliations:** 1 VISN 16/CAVHS Geriatric Research Education and Clinical Center (GRECC), 2200 Fort Roots Drive, 3J/GRECC, North Little Rock, AR 72114, USA; melinda.bopp@va.gov (M.M.B.); robersonpaulak@uams.edu (P.K.R.); Shelly.Lensing@va.gov (S.L.); dennis.sullivan3@va.gov (D.H.S.); 2 Department of Biostatistics, College of Medicine; University of Arkansas for Medical Sciences, 4301 W. Markham St. #781, Little Rock, AR 72205, USA; 3 Donald W. Reynolds Department of Geriatrics, University of Arkansas for Medical Sciences, Little Rock, AR 72205, USA

**Keywords:** nutrition assessment, nutrition status, nutritional deficiency, undernutrition, diet

## Abstract

The purpose of this study was to evaluate a multi-component method for capturing nutrient intake, which used observation, photography, and an innovative computer program. To assess reliability and accuracy, multiple responsible employees (REs) independently conducted nutrient intake assessments on simulated meals; each RE’s results relating to energy intake were compared to those from the other REs and to those obtained by pre- and post-meal weighing of the food items. System efficiency was assessed by having REs perform independent assessments on the same set of simulated meals using either the new or traditional hospital method for which the REs had to document each food item served and then find the items in a computer database–steps that were automated in the new method. Interrater reliability for energy intake estimated on clinic wards was excellent (intraclass correlation coefficient = 0.975, 95% CI 0.958 to 0.992) and there was a high level of agreement between the REs’ estimates and the true values determined by food weighing; per the method of Bland and Altman the mean difference between the two types of estimates was 0.3 kcal (95% CI, −8.1 to 8.7 kcal) with limits of agreement of −79.5 kcal to 80.1 kcal. Compared to the traditional method, energy intake assessments could be completed using the multi-component method in less than a third of the time. These results indicate the multi-component method is an accurate, reliable, and efficient method of obtaining energy intake assessments for hospitalized patients.

## 1. Introduction

Protein-energy undernutrition is a common, frequently unrecognized, and potentially serious problem among hospitalized patients [1,2,3]. When such deficits are present at admission or develop during the hospitalization, they place the patient at high risk of adverse clinical outcomes [4,5,6,7,8]. To help prevent such adverse outcomes, it is important that patients maintain a nutrient intake adequate to prevent the development of new nutritional deficits and, ideally, replete existing ones. However, this often does not occur; many patients maintain very low nutrient intakes while hospitalized [9,10]. Contributing to the problem, the healthcare team often does not recognize when a patient’s nutrient intake is inadequate [10,11,12]. For this reason, it is sometimes important to obtain precise estimates of a patient’s nutrient intake, especially when the patient is very ill or not recovering as expected [13]. Without such estimates, the healthcare team is unable to assess the patient’s nutritional risk properly and intervene proactively when necessary.

There are numerous barriers to obtaining precise estimations of a patient’s nutrient intake within the hospital; the standard process of obtaining such estimates is very labor intensive, and the staff often do not have the time or training to perform this function. As a substitute, care providers often make rapid assessments that are not accurate enough to guide clinical decision-making [14,15,16,17,18,19]. Food frequency questionnaires or 24 h recall food records completed by patients or staff also generally lack adequate reliability and accuracy for clinical use, particularly within the hospital setting [19,20,21,22]. Methods are needed that allow estimates to be more accurate with less time and effort.

In a previous paper, our group presented an innovative multi-component method for obtaining nutrient intake assessments within a United States Department of Veterans Affairs hospital [23]. We developed this innovative method as part of a large nutrition study of hospitalized older adults [8,24,25]; it combines the use of a specialized computer program, digital photography, and direct observation to provide detailed calorie count reports that include a complete listing of the total amounts of energy, proteins, fats and select micronutrients consumed by the given patient on any day of observation. In this paper, we report the results of evaluation studies of the Multi-Component Method with regards to the assessment of energy intake and discuss its strengths and limitations.

## 2. Materials and Methods

As described elsewhere [23], the computer program generates a nutritional intake assessment form (NIAF) listing the quantity and description of each food item scheduled to be served to a given patient at a given meal. This list is based on the hospital menu for the day, diet, and menu cycle selected. A responsible employee (RE) uses the NIAF as a checklist to confirm the meal tray delivered to the given patient contains all of the listed foods. If there are discrepancies, the RE edits the NIAF at the bedside so there is an exact match with what the patient actually receives. At that time, the RE also takes a photograph of the food tray. At the end of the meal, the RE takes another photo of the tray and records on the NIAF the percentage of each food item the patient consumed. The RE similarly notes food items added to or removed from the tray during the meal. Foods brought in from outside the hospital can be added to the NIAF at any time as described elsewhere [23]. Later, the RE uses the photographs to confirm his/her bedside estimations of the percentages of each food item consumed then enters the final estimates into the computer program. Once the data are entered, the computer program produces a detailed calorie count report that includes a complete listing of the total amounts of energy, protein, fats, and select micronutrients consumed by the given patient at the given meal.

### 2.1. Staff Training

Before being allowed to use the Multi-Component Method for obtaining calorie counts in a clinical setting, all RE candidates undergo a period of intense training that includes: (a) a thorough orientation to the computer program, the digital camera, and the observation protocol; and (b) multiple mentored practice sessions. At each practice session, the REs learn how to obtain accurate estimates of a patient’s nutrient intake by honing their skills taking pictures of the food trays, editing the NIAF to match the meal actually served, estimating and recording the amounts of each food item consumed during the meal, and becoming proficient with the computer program.

Initially, all of the practice sessions were conducted in the clinical laboratory using simulated patients (see Table 1). At the start of each session, each RE independently compared the items on a food tray delivered from the facility kitchen with what was listed on the NIAF printed for that meal, then edited their copy of the NIAF to make it match the food tray. After approximately 10 min, the RE trainees left the training area, and the RE trainer then weighed each individual component of the meal and recorded these weights. Next, the trainer simulated consumption of all or a part of the meal by discarding random portions of the food items on the tray. This simulation included all beverages and condiments to ensure all nutritional components were captured. While the meal was being altered, the trainer reweighed the remaining food items individually and calculated the percentage of each item’s consumption using the following formula: ((original weight − leftover weight)/original weight) × 100. When the trainer was ready, the RE trainees returned to the training area where each individual would independently estimate the percentage of each food that was ‘consumed’ (i.e., removed from the tray) and entered their estimates on their copy of the NIAF. After approximately 10 min, the trainees were instructed to enter their data into the computer and then use the menu option to calculate the amount of energy consumed by each simulated patient. The trainer did the same thing for the data based on food weight. The result from each RE was then compared to those from the other REs in a pair-wise manner and to the estimate of total energy consumed based on food weights. This allowed each RE to see how well their estimate compared that of the other REs as well as to the actual value based on weight.

The REs also conducted unblinded group learning sessions during which they repeatedly reweighed food items each time they removed another arbitrarily sized portion. This exercise supplemented the mentored training sessions in that it helped the REs further develop their visual skills against actual weights, which was especially beneficial in helping them make better estimations when dealing with certain hard to assess food items such as a stew.

As part of their overall training, the REs also learned to use the before and after meal photographs that they took. With practice, the REs were able to make relatively precise estimates of nutrient intake using only the photographs, in a manner similar to what has been reported by other investigators [26,27,28,29]. However, the REs were trained to use the photographs primarily to double check the accuracy of the NIAF that they or someone else had completed at the end of the meal. For the training, the REs were instructed to study the photos carefully after the meal was complete, the meal trays had been sent back to the kitchen, and all of the NIAFs from the meal had been filled out and were ready to be entered into the computer. The most common errors detected by reviewing the photographs in this manner were simple typographical errors (e.g., entering 100 instead of 10) and failure of the direct observer to include a food item from the tray on the NIAF. If the photograph reviewer detected differences between the estimates based on direct observation and his/her own estimates based on viewing the photos that he/she felt did not represent an obvious error, the default was to accept the estimate based on direct observation.

REs continued to participate in these training exercises, conducted multiple times each day at the breakfast, lunch and dinner meals, until they became adept at estimating nutrient intake (i.e., RE estimates generally within 15% of estimates based on weighing all food items). Once the training was completed, formal reliability and accuracy assessments of the REs’ estimates for energy intake were conducted in the laboratory and later in actual clinical settings as discussed below.

### 2.2. Reliability and Accuracy of the Calorie Counts

The Multi-Component Method was initially assessed for accuracy and inter-rater reliability by three REs who independently conducted complete calorie counts in the clinical laboratory (see Table 1, Strategy B). For these sessions, the REs worked with a trainer and used extra trays donated by the facility, as they had done for their initial training. For each evaluation day, two sample trays were provided at breakfast, lunch, and dinner (for a total of six trays). Each of the two trays from the set contained a meal from a different diet. For example, one tray would contain a full liquid diet meal and the other a meal from the regular 1800 Kcal diet menu. The REs reviewed the trays per protocol before and after the trainer weighed and removed items as described above. The REs were blinded to all measures (i.e., food weights) made by the trainer. REs evaluated 30 trays over five non-consecutive days.

After the RE trainees entered their final estimates for all meals into the computer, they used the menu option to calculate the amount of energy consumed by each simulated patient. To assess accuracy, these results were compared to the estimates of total energy consumed based on weighed food values. To assess inter-rater reliability, the RE estimates were compared to each other.

Inter-rater reliability was also assessed in the clinical setting (Table 1, Strategy C). For this assessment, six different REs participated, each performing five breakfast, lunch and dinner calorie counts (total of 15 calorie counts per RE) at the same time as, but independent from, at least two other REs. REs were initially grouped randomly although some modifications to assignments had to be made to accommodate when the REs could change from morning shift (for breakfast) to afternoon shift (to be available for dinner). All of the patients used for this evaluation were part of the larger nutrition study. Patients were selected on a given day based on their nutrition plan to ensure that a variety of diets were included [8,24,25]. For this series of inter-rater reliability tests, each RE generally performed two to four calorie counts per day but never more than three for the same meal (e.g., lunch) on a given day.

### 2.3. Dietary Staff Time Savings

A major concern for the Multi-Component Method was whether the effort expended resulted in time savings for the professional staff. To make this determination, we compared the time needed to obtain a complete calorie count using the Multi-Component Method to the time needed to obtain similar data using the traditional method used by the hospital’s dietary staff (Strategy D, Table 1). In the traditional method, an RE (usually a dietitian or dietary aid) reviewed each patient’s tray during a given meal and constructed a *de novo* hand-written listing of all of the food items served and the percentages of each that the patient consumed. The RE then used a computer program to look up each food, modified the nutrient values for serving size, and then readjusted the values for how much of that serving a patient ate before finally calculating the total calories consumed. In the larger nutrition study, this same approach had been used extensively to obtain daily calorie counts prior to the development of the new Multi-Component Method [30]. The computer program used to derive the calorie counts for the traditional method was obtained commercially (Nutritionist V^®^, 2002, The Hearst Corp, San Bruno, CA, USA). According to the manufacturer, the nutrient data used by Nutritionist V^®^ were obtained from the United States Department of Agriculture (USDA) National Nutrient Database for Standard Reference [31].

To make the time comparison, four REs each performed a series of calorie counts using the traditional method or the Multi-Component Method. All of the REs were well trained with both methods as the Multi-Component Method had only recently been placed into operation. In order to control environmental factors, we conducted the comparison trial in the training kitchen using simulated meals. It was not possible to blind the REs to the purpose of the exercise completely because we felt it important to have all of the REs use both methods in an alternating manner to obtain their calorie counts. Table 2 presents the assignment matrix used for this part of the study. As shown, the comparison study was performed using the four REs (i.e., raters), the two methods for obtaining calorie counts (traditional method and Multi-Component Method), three different days, two meals (i.e., sessions) per day, and three meals (breakfast, lunch, and dinner). For each session on each day, each RE’s time to complete each of three tasks was recorded. These three tasks were to: (1) verify what foods were provided on the pre-meal tray (and take pictures if using Multi-Component Method for the given meal); (2) verify what foods were on the post-meal tray, estimate the percentage of each consumed, and take a picture (if using Multi-Component Method); and, (3) enter all of the data into the computer program using the software for the method being employed and then calculate the total energy consumed at the given meal. At each session, half the REs completed the calorie count on the given tray using traditional method and the other half used the Multi-Component Method. On each day of the exercise, the REs evaluated a meal tray for a different meal (i.e., breakfast, lunch, or dinner) in the second session than what was used in the first to avoid contaminating the results of the second session due to what was learned during the first session. We used random assignment based on a computerized random number generator to sequence the sessions and the REs.

### 2.4. Statistical Methods

The study strategies are summarized in Table 1. For the overall assessment of accuracy (Table 1, Strategy B), it was assumed that if a given rater’s estimates were in agreement with the true values then the following formula should be true: RE estimate = slope × true value + intercept, where intercept = 0 and slope = 1. A mixed model was fit with the outcome being raters’ estimates and the independent variable being true value; a random tray effect was included to account for the clustering of raters within tray. *p*-values were computed for testing the null hypotheses that the intercept was zero (0) and the slope was one (1); a *p*-value greater than 0.05 would indicate that raters’ estimates did not significantly differ from the true value. As an indicator of the accuracy of the individual RE estimates, agreement between REs’ estimates and estimates obtained by food weighing was analyzed according to the method of Bland and Altman ignoring the clustering of raters within trays [32].

Inter-rater reliability was also assessed using two different methods. For both methods, the RE’s estimates of percentage of each food consumed were converted to an estimate of total energy consumed at the meal by entering the data into the computer program. The Intra-class Correlation Coefficient calculated using a mixed model analysis of covariance was then used to gauge reliability of these estimates. For the RE estimates obtained during the monitored training sessions during which the same raters evaluated all of the trays (i.e., Table 1, Strategy B), the model we used included fixed effects for each covariate i.e., day (5 different days) and meal (breakfast, lunch, dinner) and a random effect for each of the 30 different food trays. To assess inter-rater reliability for the RE’s estimates obtained on the clinical wards assessing actual patients (i.e., Table 1, Strategy C), the mixed model analysis of covariance was set up to account for the fact that all trays were not evaluated by all of the same REs.

To evaluate differences in total time required to obtain each calorie count according to the method used (i.e., the traditional method or the Multi-Component Method), a mixed model analysis of covariance was used. This model included fixed effects for method (traditional method vs. Multi-Component Method) and for each covariate i.e., day (three (3) different days), session (two (2) per day), and meal (breakfast, lunch, dinner) and a random effect for each RE (the four (4) REs). Two-way interactions were also investigated but none were significant, so interactions were not included in the final models. The total time for each RE to complete each calorie count was calculated as the sum of the times for each of the three calorie count tasks (as described above and in Table 1). Total time was then log transformed. In this manner, the anti-log of the difference between estimates represented percent change in the median time needed to complete each calorie count using the Multi-Component Method vs. traditional method.

All analyses were conducted using SAS Enterprise Guide v5.1 (SAS Institute Inc., Cary, NC, USA). A two-sided value of *p* < 0.05 was considered significant.

## 3. Results

### 3.1. Reliability and Accuracy of the Calorie Counts

After the training and practice sessions described above (see Table 1, Strategy C), for each RE, the average of all calorie counts differed from the average obtained by weighing the food by less than 5%, indicating there was little bias in the estimates. In addition, the rater’s estimates did not significantly differ from the true values as indicated by the relationship between the rater’s estimates and the true values being consistent with the line y = x. The intercept was estimated to be 5.5 (95% CI, −32.6 to 43.7) and did not significantly differ from 0 (*p* = 0.766), and the slope estimate was 0.99 (95% CI, 0.93 to 1.05) and did not significantly differ from 1 (*p* = 0.690).

**Figure 1:**
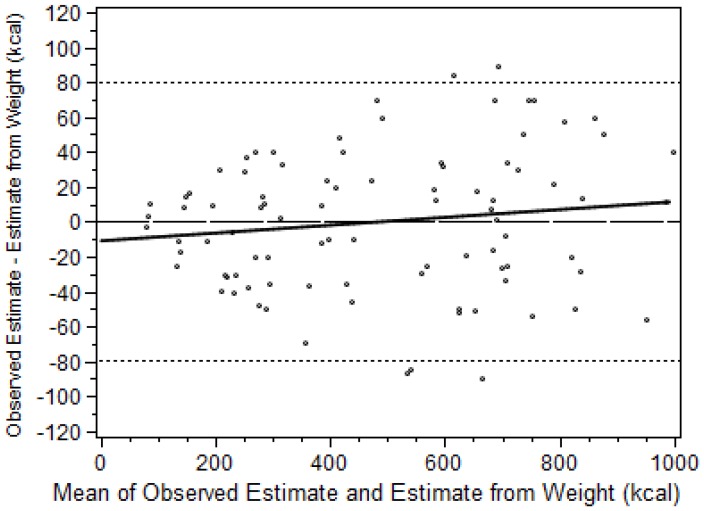
Agreement between the two methods of estimating energy consumption (RE and food weighing, *n* = 90) as depicted using the method of Bland and Altman [32]. Linear correlation between the differences and the means indicated by solid line (y = 0.02x − 10.6, *r*^2^ = 0.02, *p* = 0.20). Limits of Agreement (i.e., the mean difference in estimates ± 2SD) indicated by dotted lines.

The level of agreement between RE estimates of energy consumption and estimates based on weighing the food items is graphically depicted in a Bland-Altman plot (Figure 1) [32]; the differences between the estimate pairs were plotted against the mean of the two estimates. The mean difference between the two types of estimates was 0.3 kcal (95% confidence interval −8.1 kcal to 8.7 kcal), *p* = 0.948), indicating no significant bias. The Limits of Agreement (i.e., the mean difference in estimates ± 2SD) were −79.5 kcal to 80.1 kcal, which were narrow enough to indicate that the RE estimates are sufficiently accurate for clinical use. The differences ranged from 89 kcal to a minus 90 kcal and only five observations were outside the limits of agreement, which is consistent with the 5% that would be expected to fall outside the limits based on a normal distribution. There was no significant correlation between the differences and the means (*r* = 0.14, *p* = 0.20) indicating that the agreement of the estimates was independent of the percentage of food calories consumed.

For the RE estimates obtained during the monitored training sessions (see Table 1, Strategy B), the ICC was 0.959 (95% CI 0.936 to 0.984) indicating excellent agreement between the REs. For the RE’s estimates obtained on the clinical wards assessing actual patient meal trays (Table 1, Strategy C), the ICC was 0.975 (95% CI 0.958 to 0.992).

### 3.2. Staff Time Savings

Based on the analytic model used, it only took a median of 32.7 percent (95% CI 31.0% to 34.4%, *p* < 0.0001) as much time to obtain each calorie count using the Multi-Component Method compared to using the older standard method previously described—a time saving of 67 percent (Table 1, Strategy D). The effect of the ‘rater’ (or each RE) on this estimate was non-significant (*p* = 0.34). However, there was a significant effect of ‘meal’ type on the estimate; the time required to obtain a calorie count at breakfast was less than for lunch, which was less than for dinner.

## 4. Discussion

### 4.1. Overview

As described in this paper, the Multi-Component Method is a reliable and accurate method for efficiently producing accurate estimates of a hospitalized patient’s energy intake. However, it is important to realize that the assessment of intake remains an estimate and that accuracy will be influenced by both random and systematic error. The Multi-Component Method is designed to minimize both forms of error. When REs are estimating the percentage of a food item that has been consumed, they use a continuous scale (i.e., 0% to 100% rounded to the nearest integer), which helps to decrease the systematic error inherent in the traditional method that uses a categorical scale (e.g., 0%, 25%, 50%, 75%, 100%). The photographs help to reduce both forms of error as follows: by providing the before and after meal photographs for the RE or another individual to use to double check the completed NIAF, errors are often found, the most frequent of which are simple recording errors and failure to include a food item on the completed NIAF. When such errors go undetected, the accuracy of the resulting estimation of intake is going to be affected. Of greater importance, the photographs allow a supervisor or other quality control specialist to monitor random RE assessments, which help to prevent rater drift. Rater drift is the deterioration in the quality of a rater’s assessments over time [33]. It can develop for a number of reasons including raters forgetting their training or becoming bored with their job and losing motivation to do it correctly [33]. Each of these components worked symbiotically to increase the accuracy of the RE’s assessment.

As described in the methods, the computer program developed for the Multi-Component Method produces a detailed listing of the micro- and macro-nutrient composition of the food that the patient consumed [23]. This report is generated using the program’s food composition database, which was based on a USDA food nutrient database and included all of the food items that are used by the hospital’s dietary service [34,35]. Additional food items can be added easily. When conducting a nutrient intake assessment, the RE need only record the percentage of each food item served that is consumed. When the RE’s estimates are entered into the computer, the program automatically calculates the micro- and macro-nutrient composition of the food that the patient consumed. In this paper, the reliability and accuracy of the resulting energy intake estimates are described. Although in this study the reliability and accuracy of the output with regards to the specific macro-nutrient content of the food consumed (i.e., protein, fat, or carbohydrate content) was not examined, it is likely that the outcome of such analyses would be similar to those presented in this paper for energy consumption. However, this needs to be verified in a separate study.

### 4.2. Strengths

Each component of the Multi-Component Method contributes unique advantages to the process as a whole. The computer program supports the REs’ direct observations and photography, and is a powerful tool for streamlining the process of producing a detailed calorie count. The calorie count report is very detailed and includes both the total calories served as well as the total calories (i.e., energy) consumed by the given patient at the given meal (or for the entire day). The report provides similar statistics for protein, fats (saturated and unsaturated), carbohydrates and multiple micronutrients (data not shown). The Multi-Component Method eliminates the time-intensive process of calculating these values by hand. Further, the Multi-Component Method makes it possible for clinicians to have real time data concerning their patients’ ongoing nutrient needs. When clinicians are aware a patient’s energy intake is inadequate, they can mount a multi-professional response to address the problem in a timely manner. Such a rapid response may help prevent the patient from developing serious nutritional deficits that can complicate recovery. The computer program’s interface enhances this communication by allowing users at multiple levels to interact with the computer program and each other with ease, regardless of profession or skill level. The ability to employ various types of employees for data collection helps to control costs.

The computer program allows each menu or meal plan to be edited easily thus allowing for rapid accommodation to temporary or permanent changes to the hospital’s dietary menu. This enhances the accuracy of the calorie count without an extensive reprogramming effort. This is an important aspect of the program because institutional meal plans change based on food availability or season. The program also decreases the time needed to determine the nutrient content of a meal and of the food actually consumed by the patient. Although non-institutional food items brought in from outside the hospital must still be added to the computer program, this process is also completed easily. Once a non-institutional food item is entered into the computer program, it is there for future use. The computer program also uses a very accurate source of nutrient information specifically matched to the hospital dietary menu, as described earlier. This eliminates errors that can be introduced by using commercial programs to look up every food item or the more ad hoc approach of using internet searches, which may be inconsistent, resulting in errors in the nutrient analysis. The multi-component method also promotes inclusion of food provided during snacks or family visits. The computer program ensures a comprehensive assessment of total nutrient intake on any given day and allows for rapid evaluation of the impact of new nutrition plans.

The NIAF generated from the computer program template helps the RE document what a patient actually receives for each meal, and the RE may edit the NIAF based on observation of the tray. The computer program also promotes continuity of observations by facilitating handoff from one RE to the next when conducting a calorie count. For example, a night shift employee might begin the breakfast count, but a day shift RE can finish the count with a lower probability of error since the NIAF and “before” photo taken on the night shift will inform the day shift RE’s calculations. Particularly when the NIAF is on a laptop, detailed calorie counting becomes a fluid process.

### 4.3. Limitations

We have also found limitations in our process. The initial investment for photographic equipment, technological support, and training personnel may be challenging with already strained budgets. Downloading photos and placing them in the correct electronic files requires a daily time investment, and additional time is required to ensure the computer program remains current with seasonal changes in the hospital menus or the addition of new foods. Labor required to perform these latter two tasks is not captured in our time assessment. Additional employee time is required to educate the patients and families about the need for the calorie count, which is useful in recruiting their assistance for accuracy. Patients and families may also question why a RE is taking pictures at mealtime, so it is critical to explain the multi-component method and the need for detailed nutrition information clearly.

There are also necessary tasks the method does not eliminate. The RE must be on the unit to monitor food alterations during the meal; a direct observation is the most effective way to capture this information. Food brought to the patient by family members necessitates that a RE enter these foods onto the NIAF. Finally, staff training and verification of quality remains a constant process, although the inclusion of photography in our process facilitates quality control. On an ongoing basis, a second RE can periodically compare the digital pictures with the data entered into the nutrition program. When the estimates of nutrient consumption made by the second RE based on the photographs differs from the original estimates by greater than a set threshold (e.g., 15%), the potential error can be flagged. When the rate of potential errors reaches a set threshold (e.g., 5%), additional training can be scheduled for the REs to improve their technique.

A significant limitation in evaluating any dietary intake methodology is the absence of a “gold standard” for the assessment of energy intake. Since validation studies are defined as “a study conducted to compare a dietary assessment instrument to a reference” [36], it is not possible to establish true validity. In the absence of such an accepted reference, we relied on food weights to establish the accuracy of the Multi-Component Method, a method previously used by numerous studies [37,38,39,40].

## 5. Conclusions

Evaluation of this Multiple Component Method clearly demonstrates it is a reliable and accurate technique for obtaining assessments of patient energy intake in a hospital setting. The method provides a detailed accounting of energy intake while saving significant time for the healthcare team.

## Figures and Tables

**Table 1:** Study design.

Strategy	Location	Sample Details	Method
A: Initial Training: in some sessions REs * were blinded to weight. Results not recorded	Clinical Laboratory	Variable number of trays; All REs	Trainer simulated meals by removing food and weighed remaining items; raters observed and photographed trays pre- and post then estimated consumption; Trainer compared RE estimates of energy consumption to estimates based on weight change
B: Initial Accuracy and Reliability testing	Clinical Laboratory	Three blinded RE raters; same 30 trays evaluated by each over five days (Total 90 assessments)	Same as for training (Strategy A). Compare RE estimates to estimates based on weights (Accuracy) and matched RE estimates to each other (Reliability)
C: Clinical Reliability assessment	Actual Clinical Setting	Each of 6 blinded REs assessed 5 breakfast, 5 lunch, and 5 dinner trays independent from but at the same time as at least 2 other REs for a total of 90 assessments. REs initially grouped randomly	Intra-class Correlation Coefficient using a Mixed Model Analysis of Covariance; fixed effects for meal and day, random effect for each individual food tray
D: Time Savings: Compare time to complete calorie counts using the automated Multi-Component Method and traditional hospital methods	Clinical Laboratory Setting	Four REs assigned to new or traditional method in alternating manner; recorded total time to complete calorie count for each RE for each session. See Table 2 for Assignment Matrix	Mixed Model Analysis of Covariance with fixed effects for method and for each covariate; random effect included for each RE

* RE = Responsible Employee (see text for details). Trainer: an assistant who helped with training and evaluation studies.

**Table 2:** Assignment matrix and times for the time savings analysis.

Rater (RE)	Method	Day	Session	Meal	Time (in Seconds)		
					Task 1 Pre-Meal	Task 2 Post-Meal	Task 3 Data Entry
1	MCM	1	1	Dinner	156	129	198
2	TM	1	1	Dinner	436	154	946
3	TM	1	1	Dinner	517	72	966
4	MCM	1	1	Dinner	228	101	209
1	TM	1	2	Lunch	498	89	906
2	MCM	1	2	Lunch	174	142	160
3	MCM	1	2	Lunch	186	184	179
4	MCM	1	2	Lunch	411	274	828
1	MCM	2	1	Breakfast	120	120	180
2	MCM	2	1	Breakfast	84	101	178
3	TM	2	1	Breakfast	420	180	740
4	TM	2	1	Breakfast	312	108	780
1	TM	2	2	Lunch	396	174	792
2	TM	2	2	Lunch	468	234	852
3	MCM	2	2	Lunch	138	108	246
4	MCM	2	2	Lunch	150	174	162
1	TM	3	1	Breakfast	96	132	186
2	MCM	3	1	Breakfast	420	159	660
3	TM	3	1	Breakfast	91	112	203
4	MCM	3	1	Breakfast	374	122	726
1	TM	3	2	Dinner	547	172	1049
2	MCM	3	2	Dinner	208	121	259
3	TM	3	2	Dinner	547	142	968
4	MCM	3	2	Dinner	189	101	199

Multi-Component Method = MCM; TM = Traditional Method; Pre-Meal Tasks = comparing meal to NIAF and taking pre-meal photo if using MCM; Post-Meal Tasks = estimation of meal consumption based on observation and post-meal photo if using MCM; Data Entry = using either MCM or TM to evaluate nutrients based on consumption observations and photos if using MCM.
